# Silicon modifies leaf nutriome and improves growth of oak seedlings exposed to phosphorus deficiency and *Phytophthora plurivora* infection

**DOI:** 10.3389/fpls.2023.1265782

**Published:** 2023-08-29

**Authors:** Igor Kostic, Nina Nikolic, Slobodan Milanovic, Ivan Milenkovic, Jelena Pavlovic, Ana Paravinja, Miroslav Nikolic

**Affiliations:** ^1^ Laboratory of Plant Nutrition, Institute for Multidisciplinary Research, University of Belgrade, Belgrade, Serbia; ^2^ Faculty of Forestry, University of Belgrade, Belgrade, Serbia; ^3^ Faculty of Forestry and Wood Technology, Mendel University in Brno, Brno, Czechia

**Keywords:** combined stress, ionomics, nutrient crosstalk, *Quercus robur*, root architecture

## Abstract

Beneficial effects of silicon (Si) on plants have primarily been studied in crop species under single stress. Moreover, nutrient acquisition-based responses to combination of biotic and abiotic stresses (a common situation in natural habitats) have rarely been reported, in particular in conjunction with soil amendments with Si. Pedunculate oak (*Quercus robur* L.), one of the ecologically and economically most important tree species in Europe, is facing a severe decline due to combined stresses, but also problems in assisted regeneration in nurseries. Here, we studied the effect of Si supply on the leaf nutriome, root traits and overall growth of 12-weeks-old oak seedlings exposed to abiotic stress [low phosphorus (P) supply], biotic stress (*Phytophthora plurivora* root infection), and their combination. The application of Si had the strongest ameliorative effect on growth, root health and root phenome under the most severe stress conditions (i.e., combination of P deficiency and *P. plurivora* root infection), where it differentially affected the uptake and leaf accumulation in 11 out of 13 analysed nutrients. Silicon supply tended to reverse the pattern of change of some, but not all, leaf nutrients affected by stresses: P, boron (B) and magnesium (Mg) under P deficiency, and P, B and sulphur (S) under pathogen attack, but also nickel (Ni) and molybdenum (Mo) under all three stresses. Surprisingly, Si affected some nutrients that were not changed by a particular stress itself and decreased leaf Mg levels under all the stresses. On the other hand, pathogen attack increased leaf accumulation of Si. This exploratory work presents the complexity of nutrient crosstalk under three stresses, and opens more questions about genetic networks that control plant physiological responses. Practically, we show a potential of Si application to improve P status and root health in oak seedlings, particularly in nurseries.

## 1 Introduction

The pedunculate oak (*Quercus robur* L.) is the most abundant deciduous tree species in Europe and one of the most ecologically and economically significant trees in the region (Eaton et al., 2016). Over the past four decades, there has been a consistent and concerning trend of oak forest deterioration reported in numerous European countries (Sonesson and Drobyshev, 2010; Denman et al., 2014; Machacova et al., 2022). The decline of oak forests is a complex phenomenon in which different biotic and abiotic factors interact simultaneously (Thomas et al., 2002). Species in the oomycete genus *Phytophthora* are considered to be one of the most important biotic factors responsible for tree mortality (Jung et al., 2000; Jönsson et al., 2005). Hemibiotrophic *Phytophthora plurivora* T. Jung & T.I. Burgess can cause serious root damage in mature trees in natural habitats, and particularly in seedlings grown in greenhouses and nurseries (Jung et al., 2016). Control measures against *Phytophthora* infection include prevention (e.g., usage of uninfested water and soil and healthy plant material) or/and application of fungicides, which negatively affect the environment and in long-term may lead to host resistance.

Low phosphorus (P) supply was shown to increase the severity of fungal and bacterial infections in several crop species (reviewed by Tripathi et al., 2022); very recently, a more complex view of plant-microbe interactions under P limitation, including molecular explanations has been provided (Paries and Gutjahr, 2023). For instance, P deficiency can induce jasmonate signalling pathway (Khan et al., 2016), which is also a critical mediator of the plant defence responses to pathogen attack (Li et al., 2022). Mature oak trees can rely on ectomycorrhiza for P acquisition, which also provides some protection against fungal root pathogens (Bennett et al., 2017). However, young seedlings in nurseries are vulnerable to P deficiency, where they often fail to achieve adequate mycorrhization (Southworth et al., 2009).

The architecture of the root system, in particular the length of thin roots and their surface area, is closely related to the uptake of mineral nutrients from soil (James et al., 2020). Alterations in any of the processes that mobilize nutrients and other mineral elements in the rhizosphere and transport them from root to shoot could potentially affect the elemental composition of plants, also known as the ionome. The plant ionome is considered a multivariable signature of a plant physiological state (Baxter et al., 2008). Over the past two decades, ionomics has been used to identify gene networks that control the *in planta* elemental homeostasis in environmental adaptations (reviewed by Huang and Salt, 2016). However, the use of ionomics in examining plant responses to pathogens has rarely been employed. On the other hand, the existing findings on the leaf ionome changes in relation to P deficiency still present some inconsistencies. Moreover, the responses to these two stresses in conjunction with the application of silicon (Si) amendments have been extremely rarely reported.

Though Si is not considered a plant nutrient, its beneficial effects in overcoming various biotic and abiotic stresses, including P deficiency and fungal infections, are well documented in agricultural crops (reviewed by Wang et al., 2017; Pavlovic et al., 2021). However, the effect of this beneficial element on plants simultaneously exposed to both biotic and abiotic stress what is a common situation in natural habitats (Teshome et al., 2020) has almost not been studied, particularly not on spontaneously growing tree species (Cooke and Leishman, 2016). Present study aimed to evaluate the effect of Si addition on overcoming growth constraints caused by P deficiency and *P. plurivora* infection in pedunculate oak and to examine the thereby induced changes in the nutrient profile, also called the nutriome. Here, we analysed: biomass accumulation, root damage, root traits (total root volume and surface, and length and surface of thin roots) and leaf mineral concentrations of nutrients [nitrogen (N), P, potassium (K), sulphur (S), calcium (Ca), magnesium (Mg), iron (Fe), manganese (Mn), boron (B), zinc (Zn), copper (Cu), molybdenum (Mo), and nickel (Ni)] and Si in the 12-weeks-old oak seedlings exposed to these two stresses and their combination.

## 2 Materials and methods

### 2.1 Soil properties

The experimental soil was a Regosol with clayey texture (22% silt, 49% clay, and 29% silt), pH (in H_2_O) 6.22, 0.57% CaCO_3_, 23 cmol_c_ kg^−1^ CEC, 1.2% organic matter, 0.18% total N, 0.17% total Sand very low in total (HNO_3_-extractable) P (206 mg kg^−1^). The available nutrients shown in **Table 1** correspond to 4.5 kg P ha^−1^, 363 kg K ha^−1^, 7500 kg Ca ha^−1^, 1995 kg Mg ha^−1^, 0.90 kg B ha^−1^, 10.2 kg Cu ha^−1^, 129 kg Fe ha^−1^, 112 kg Mn ha^−1^, 2.9 kg Zn ha^−1^, 0.06 kg Mo ha^−1^, and 6.9 kg Ni ha^−1^ in top soil (20 cm). The soil concentration of available Si, extracted with 0.01 M CaCl_2_, was 39.7 mg kg^−1^, corresponding to 119 mg Si ha^−1^ in top soil. Therefore, the only limiting factor for plant growth was P deficiency.

**Table 1:** Concentrations of available nutrients in the experimental soil.

Nutrient	Extraction method	mg kg^−1^
P	NaHCO_3_ (Olsen)	1.5
K	NH_4_CH_3_CO_2_	121
Ca	NH_4_CH_3_CO_2_	2500
Mg	NH_4_CH_3_CO_2_	665
B	Sorbitol	0.30
Cu	DTPA	3.4
Fe	DTPA	42.9
Mn	DTPA	37.2
Zn	DTPA	0.98
Mo	DTPA	0.02
Ni	DTPA	2.3

### 2.2 Experimental design, plant material and growth condition

The full factorial randomized experimental design included combinations of three factors with two levels each (2 × 2 × 2): soil P supply (–/+P), infection with *Phytophthora plurivora* (–/+Phyt) and Si addition (–/+Si), totalling eight treatments: –P–Phyt–Si; –P+Phyt–Si; –P–Phyt+Si; –P+Phyt+Si; +P–Phyt–Si; +P+Phyt–Si; +P–Phyt+Si, and +P+Phyt+Si; each treatment was run in 10 replicates. The treatment without experimental stresses and without Si addition (+P–Phyt–Si) was referred to as a control. Phosphorus (+P) was applied as KH_2_PO_4_ in the amount of 180 mg P kg^−1^ dry soil, corresponding to a field application of about 260 kg P ha^−1^. The soil was thoroughly mixed with the amendment prior to filling the pots. Silicon (+Si) was added as monosilicic acid (H_4_SiO_4_) in the amount of 300 mg Si kg^−1^ dry soil, corresponding to a field application of about 450 kg Si ha^−1^. The 50 mM stock solution of H_4_SiO_4_ (pH 2.5) was freshly prepared by passing Na_2_SiO_3_ through a plastic column filled with cation-exchange resin (Pavlovic et al., 2013) and each pot received 79 ml of the stock solution. The soil pH reached equilibrium (about 6) after the addition of H_4_SiO_4_ within 24 h.

Acorns of the pedunculate oak (*Quercus robur* L.) were thoroughly rinsed with distilled water and germinated on moist filter paper in an incubator at 28°C. After germination, two uniform acorns were planted in the plastic pots (volume 576 cm^3^) filled with 370 g of the dry soil, and then watered to achieve about 70% of the field water capacity. In half of the pots, two plastic tubes were inserted into the soil to facilitate the addition of *Phytophthora plurivora* inoculum without causing any mechanical damage of the roots. The cotyledons of the seedlings were removed at emergence to prevent the transfer of nutrients from cotyledons to the young plant. Five days after seedling emergence, each pot was thinned to one plant. Plants were grown under controlled environmental conditions in a growth chamber with a light/dark regime of 16/8 h, the photon flux density of 300 μmol m^−2^ s^−1^ at plant height provided by led panels (Apollo 8, Cidly Co., Ltd., Shenzhen, China), the temperature of 26 ± 2°C and relative air humidity of about 70%. Plants were grown for 12 weeks, i.e., 8 weeks before the inoculation, and 4 weeks until the first disease symptoms (tissue necrosis, chlorosis, and reduced leaf size) appeared on leaves of the inoculated seedlings. During the entire experimental period, the oak seedlings were irrigated to 70% of the field water capacity, and the physical position of the pots was changed randomly.

At the end of experiment, the seedlings were carefully excavated, soil removed from roots by shaking, divided into roots and shoots and washed thoroughly with tap water and rinsed with deionized water. Roots were subjected to analyses of the selected traits, whereas leaves were oven dried at 65°C for 72 h and weighted.

### 2.3 Root inoculation with *Phytophthora plurivora*

Briefly, inoculum of *Phytophthora plurivora* (GenBank: KF234666) isolate, was prepared using fine vermiculite, millet seeds, and V8^®^ vegetable juice according to Jung et al. (1996). In half of the pots containing 8-week-old oak seedlings, approximately 7.5 g of inoculum per pot was carefully added to the voids created after removing the plastic tubes from the soil. After the inoculation, the pots were kept for 72 h submerged in distilled water. At the end of the experiment, the establishment of the root infection by *P. plurivora* was confirmed by plating small pieces from the necrotic lesions and fine roots on selective V8A-PARPNH medium (Jung et al., 1996).

### 2.4 Measurement of the root traits

Roots were visually inspected using binocular for the estimation of the share of the surface affected by the symptoms caused by *P. plurivora* infection (i.e., % of root surface covered with lesions and necrosis). Subsequently, the roots were scanned (Epson Expression 12000XL Photo Scanner) and analysed by WinRHIZO® (ver. 2017) software (Regent Instruments Inc., Quebec, Canada). After oven drying at 65°C, dry weight of fine and main roots was recorded. The following root traits were calculated: total root surface area, total root volume, length of thin roots and projected area of thin roots. Thin roots were those with diameter of 1-2 mm.

### 2.5 Determination of leaf mineral elements

The pulverized dried leaf material (0.2 g) was microwave digested with 3 ml concentrated HNO_3 _+ 2 ml H_2_O_2_ for 1 h in a microwave oven (ETHOS EASY, Milestone Srl, Sorisole, Italy). The concentration of Si was determined by the inductively coupled plasma optical emission spectrometry (ICP-OES; Spectro Genesis EOP II, Spectro Analytical Instruments GmbH, Kleve, Germany) equipped with Spectro hydrofluoric acid (HF) resistant sample introduction system after incubation of the digested samples with 1 ml concentrated HF for 12 h. The concentrations of P, K, Ca, Mg, B, Fe, Cu, Mn, Zn, Mo, and Ni in the digested samples were determined by the inductively coupled plasma mass spectrometry (ICP-MS; Agilent 8900 ICP-QQQ, Agilent Technologies, Inc., Santa Clara, CA, USA). The concentrations of N and S were determined by direct combustion of the dried leaf samples in a Vario Microcube CHNS analyser (Elementar Analysensysteme GmbH, Hanau, Germany). The certified reference material (GBW10015 Spinach; Institute for Geophysical and Geochemical Exploration, Langfang, China) was used to evaluate the precision and accuracy of the mineral analyses.

### 2.6 Statistical analyses

The effects of treatment factors (P supply, inoculation with *P. plurivora*, and addition of Si) on the measured plant variables (total dry biomass, degree of root damage, root/shoot ratio, total root volume, total root projected area, length of thin roots, surface area of thin roots as well as concentrations of mineral elements in leaves) were analysed by a factorial ANOVA model with interaction term (STATISTICA 6 software, StatSoft Inc., Tulsa, OK, USA), using the confidence level of α=0.05, and a conservative Tukey’s *post hoc* test. Significant statistical interaction (P × Phyt × Si) can be interpreted as a significant effect of Si addition on modifying the joint effect of different combinations of the abiotic and the biotic stress on leaf nutrient concentration. ANOVA t-value (i.e., the size of the observed difference scaled in standard errors) is presented as a proxy of the effect strength. Differences of leaf nutriome caused by different treatments were analysed by a nonparametric Multi-Response Permutation Procedure (MRPP, Mielke and Berry, 2001) using the Euclidean distance measure. Prior to MRPP, the leaf elemental concentrations were adjusted to standard deviate, to bring them to an equal footing. Simultaneous grouping of treatment combinations and leaf concentrations of plant essential mineral elements was done by two-way cluster analysis (hierarchical clustering: Euclidean distance, Ward’s linkage, no relativization, and matrix coding as relative values by matrix); data matrix contained statistically significant relative changes (in % of the control, +P–Phyt–Si) of leaf elemental concentration. The presented “information remaining” axes in a dendrogram are rescaled Wishart’s objective functions. Statistically significant differences in elemental concentrations between the control and other treatments were established by a series of t-tests. For visualization of the multivariate changes, free ordination of samples defined by leaf elemental concentration was done by Principal Component Analysis (PCA), using correlation cross-product matrix (inbuilt relativization). The number of significant PCA axes (i.e., principal components) to be interpreted was determined after Monte Carlo randomization test with 999 runs. To assess the relative contribution of Si addition to “explaining” the encountered differences in leaf nutriomes at each of the two contrasting levels of P supply, the SumF analysis (Warton and Hudson, 2004) was applied to a two-way factorial model. This is a permutation-based multivariate analysis of variance, based on pooling univariate F-statistics (which do not depend on any linear scaling). Dissimilarity matrix of leaf nutriome was calculated using Gower’s distance on the data matrix containing untransformed mean values for each mineral nutrient and for Si (14 mineral elements in total) in each treatment combination. Gower’s is a proportional city-block distance measure based on absolute difference relativized by a range of variable values. The PC-ORD 7.11 software (MjM Software Design, Gleneden Beach, OR, USA) was used for all the multivariate analyses, with the protocols described in McCune et al. (2002).

## 3 Results

### 3.1 Effect on plant fitness

**Figure 1:**
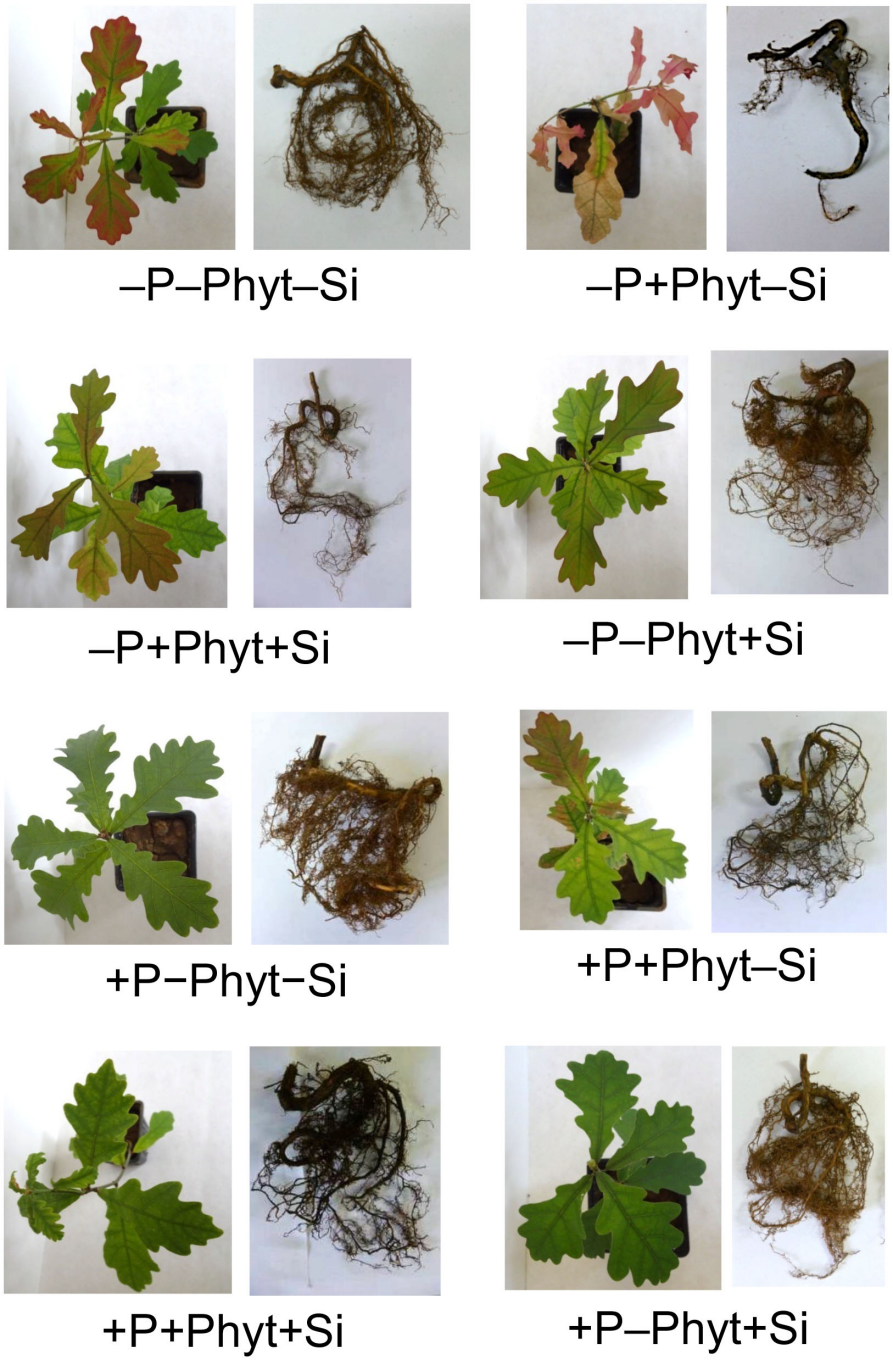
The effect of treatment factors (P supply, *Phytophthora plurivora* root infection and Si addition) on the visual appearance of 12-week-old oak seedlings.

The treatment factors (P supply, root infection by *P. plurivora*, and the addition of Si) markedly affected the growth and visual appearance of 12-week-old seedlings of pedunculate oak (**Figure 1**). The addition of Si had no visible influence only on distinguishing +P–Phyt+Si from +P–Phyt–Si treatments. The most prominent retardation of both root and shoot overall growth as well as the most intense stress symptoms on leaves (tissue necrosis, internerval chlorosis, purple coloration) and on roots (necrotic lesions) were observed when oak seedlings were subjected to both a biotic and an abiotic stress in the absence of Si application (–P+Phyt–Si; **Figure 1**).

**Figure 2:**
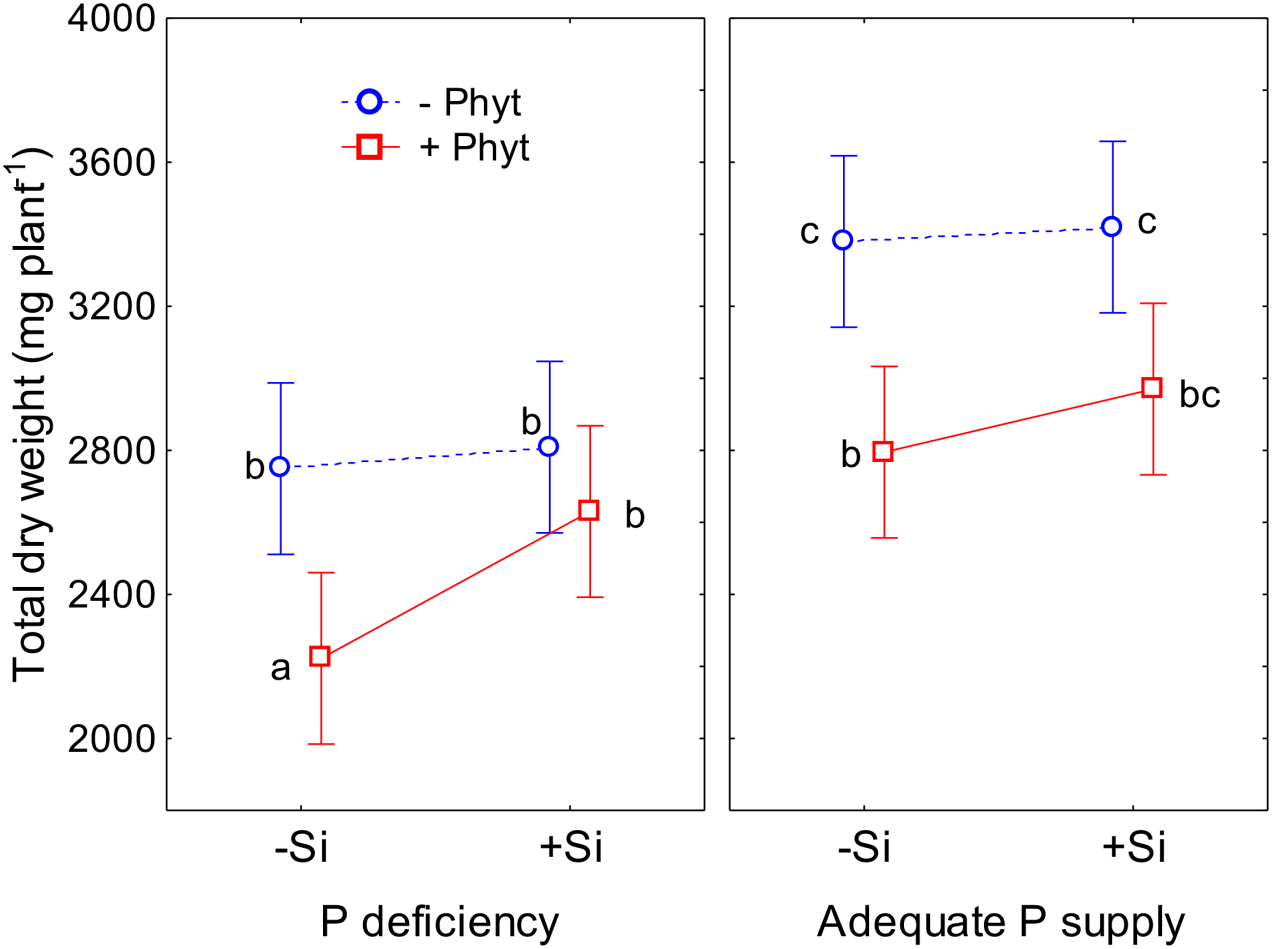
The effect of phosphorus (P) supply, *Phytophthora plurivora* root infection and silicon (Si) addition on total dry weight of 12-weeks-old oak seedlings. Statistical interaction P × Phyt × Si (F(1,72)=0.40, p=0.53) is shown. The same letters indicate no statistically significant differences (α=0.05) among the treatment means (10 plants per treatment) according to the Tukey test following 3-way ANOVA. Vertical bars denote 95% confidence intervals. Full model: R^2^adj. 0.47, F(7,72)=10.7, p <0.0000001.

All the three experimental factors (the level of P supply, *P. plurivora* root infection, and the addition of Si) were shown to be nominally significant predictors of the total plant dry weight (DW; **Table S1**). The modifying effect of Si supplementation on the growth of oak seedlings exposed to an abiotic and a biotic stress is presented in **Figure 2**. At an adequate P supply to the non-infected plants, Si did not affect biomass accumulation, while only marginal (statistically insignificant at α=0.05) improvement was observed in the infected seedlings (**Figure 2**). Nevertheless, in –Phyt –Si plants P deficiency significantly decreased total biomass by about 23%. Furthermore, at adequate P supply and no Si addition, root infection *per se* significantly decreased biomass accumulation by about 17%. The largest growth suppression by about 34% (relative to the control: adequate P supply, no infection by *P. plurivora*, no Si addition), was observed in the combination of both stresses when no Si was added. The addition of Si significantly improved the total biomass production in the infected P deficient oak seedlings by about 18%, bringing it to the level of the non-infected ones. Thus, Si supplementation of the plants exposed to both stresses (–P+Phyt+Si) recovered the DW accumulation to the level of the infected plants at adequate P supply (+P+Phyt–Si; **Figure 2**), what was also reflected on visual symptoms on leaves and roots (**Figure 1**). On the other hand, no significant effect of any of the three tested factors on root/shoot ratio was detected during the course of this experiment.

**Figure 3:**
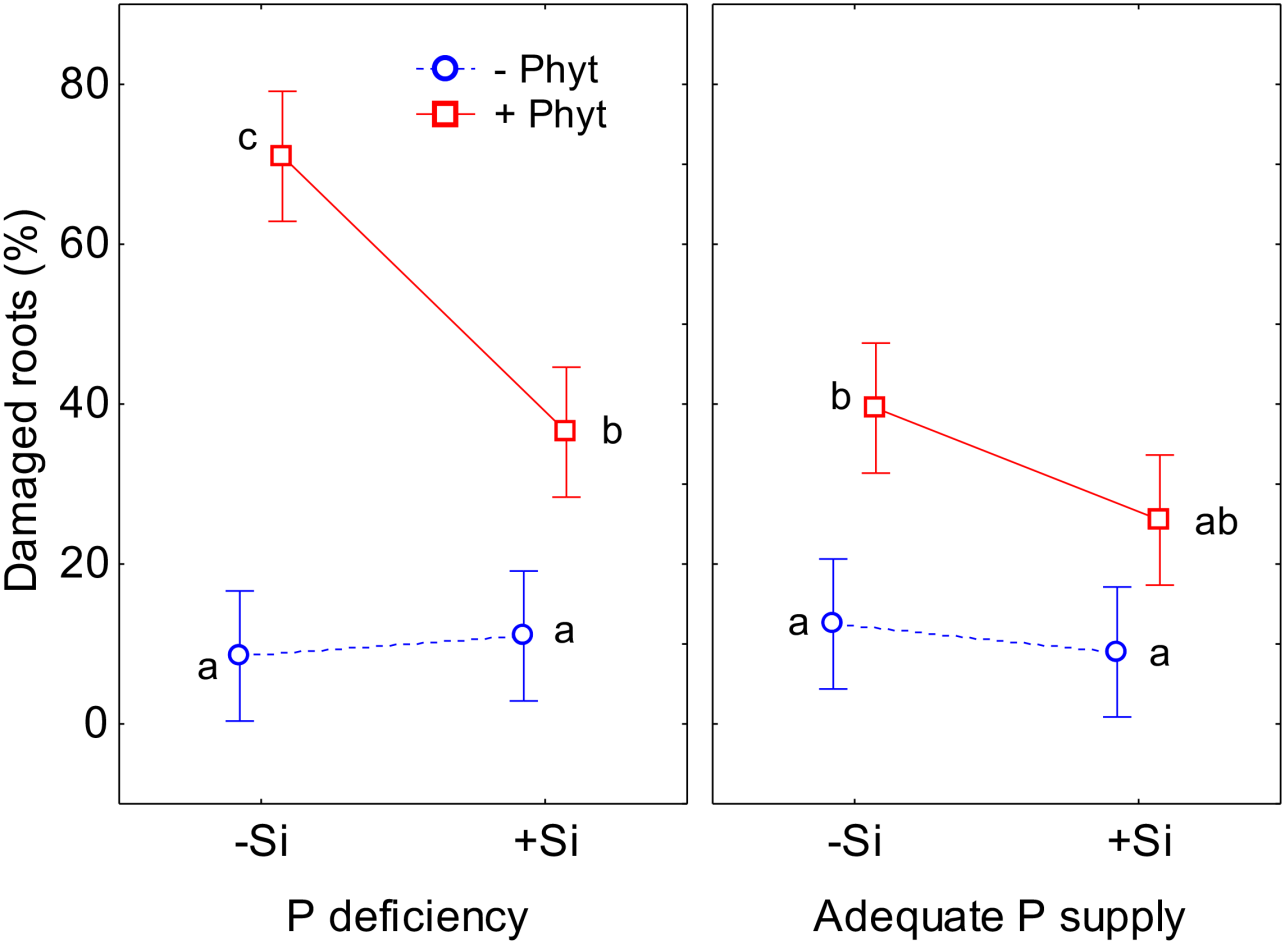
The effect of phosphorus (P) supply, *Phytophthora plurivora* root infection and silicon (Si) addition on the root health status of pedunculate oak seedlings. Statistical interaction P × Phyt × Si (F(1,72)=5.3, p=0.025) is shown. The same letters indicate no statistically significant differences (α=0.05) among the treatment means (10 plants per treatment) according to the Tukey test following 3-way ANOVA. Vertical bars denote 95% confidence intervals. Full model: R^2^adj. 0.71, F(7,72)=28.5, p<0.0000001.

Furthermore, the three treatment factors significantly affected the percentage of the damaged root surface caused by *P. plurivora* (visually observed necrotic lesions; **Table S2**; see also **Figure 1**). The addition of Si had, as expected, no effect on root health in plants not inoculated with *P. plurivora*. The inoculation with the pathogen resulted in a substantial increase in the observed root damage (percentage of root surface with necrotic lesions) in adequately P-fed plants, approximately 3.2 times higher than the control (+P–Phyt–Si). This damage was only marginally ameliorated by Si addition (+P+Phyt–Si *versus* +P+Phyt+Si; **Figure 3**): not significantly by the full ANOVA model at α=0.05, but significantly according to the t-test (t-value 2.1, p=0.0499). On the other hand, P deprivation increased the root damage by about 80% when no Si was added. The addition of Si to seedlings deficient in P and infected by *P. plurivora* very prominently ameliorated the root damage, lowering the extent of the necrotic lesions by about 2 times (**Figure 3**), thereby bringing the level of the damage to the one in adequately P supplied plants. Thus, under the combination of a biotic and an abiotic stress, Si application improved the root health status to the level of damage caused by the biotic stress only (**Figure 3**).

### 3.2 Effect on root phenome

**Figure 4:**
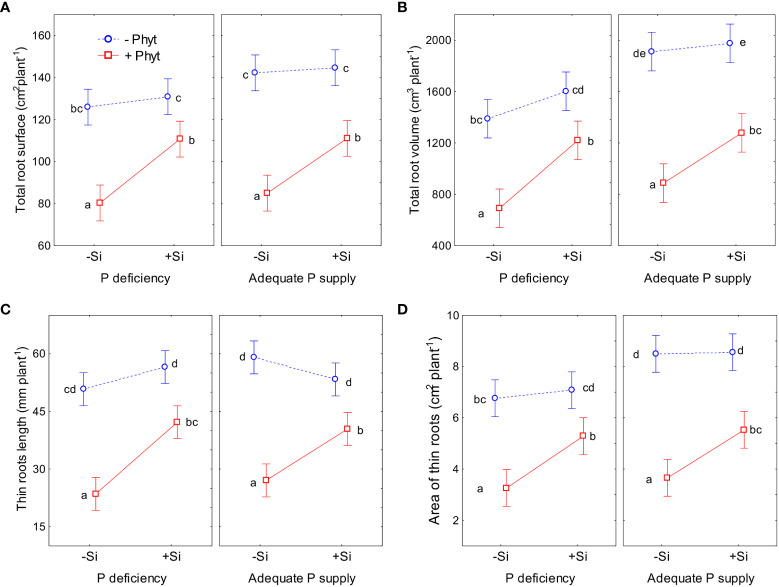
The effect of phosphorus (P) supply, *Phytophthora plurivora* root infection and silicon (Si) addition on the selected root phenes. Statistical interactions P × Phyt × Si are shown. Total root surface area: F(1,72)=0.02, p=0.89; full model R^2^adj. 0.73, F(7,72)=32.1, p<0.0000001 **(A)**; Total root volume: F(1, 72)=0.0003, p=0.095; full model R^2^adj. 0.76, F(7,72)=36.3, p<0.0000001 **(B)**; Length of thin roots: F(1,72)=1.05, p=0.31; full model R^2^adj. 0.77, F=(7,72)=38.2, p<0.0000001 **(C)**; Projected area of thin roots: (F1,72)=0.009, p=0.092; full model R^2^adj. 0.73, F(1,72)=31.1, p<0.0000001 **(D)**. The same letters indicate no statistically significant differences (α=0.05) among the treatment means (10 plants per treatment) for each phene, according to the Tukey test following each 3-way ANOVA. Vertical bars denote 95% confidence intervals.

The effect of P status, *P. plurivora* root infection and Si addition on the selected root morphological traits are shown in **Figure 4** (see also **Figure 1**). The details of the ANOVA analyses are given in **Tables S3**-**S6**. The pattern of change of all the four examined phenes was essentially the same and did not substantially differ at low *vs* adequate P supply. Overall, P deficiency *per se* did not stimulate root growth in 12-weeks-old oak plants; the growth decrease induced by the lack of P was detected in 2 out of 4 phenes (**Figures 4B, D**; **Tables S4**, **S6**). The infection with *P. plurivora* had the strongest effect in decreasing the measured root traits; this reduction of root growth was, on average, by 40% in the per plant total projected root surface area (**Figure 4A**), by 53% in the total root volume (**Figure 4B**), by 54% in the length of thin roots (**Figure 4C**), and by 57% in the projected surface area of thin roots (**Figure 4D**). Moreover, *P. plurivora* tended to cause stronger root growth suppression in adequately P fed plants than in the P deficient ones (negative coefficient of statistically significant interaction P × *P. plurivora* infection; see **Tables S3**, **S4**, **S6**). Compared to +P–Phyt–Si treatment as a control, the strongest suppression of all the 4 measured root traits was observed in the infected plants not treated with Si. The ameliorative effect of Si addition on the root phenes was detected only in the infected plants, while there was no response to Si addition in healthy plants.

The application of Si increased the total root surface of the infected plants by 34% (**Figure 4A**). In the same line, the addition of Si increased total root volume of the oak seedlings infected by *P. plurivora* by on average 58% (**Figure 4B**). In the infected plants supplied with Si, the length of thin roots was increased by 63% as compared to the infected plants without Si addition (**Figure 4C**). Besides, it was possible to statistically detect the ameliorative effect of Si supply on P deficiency for this phene (interaction P deficiency × Si addition; **Table S5**). Across both healthy and infected 12-weeks-old oak seedlings, the addition of Si to P-deficient plants increased the length of thin roots by about 32%, recuperating it to the level of P-sufficient ones. Finally, the projected surface area of thin roots in Si-supplied infected seedlings was by 56% higher than in diseased seedlings without Si supply (**Figure 4D**).

### 3.3 Effect on leaf nutriome

**Figure 5:**
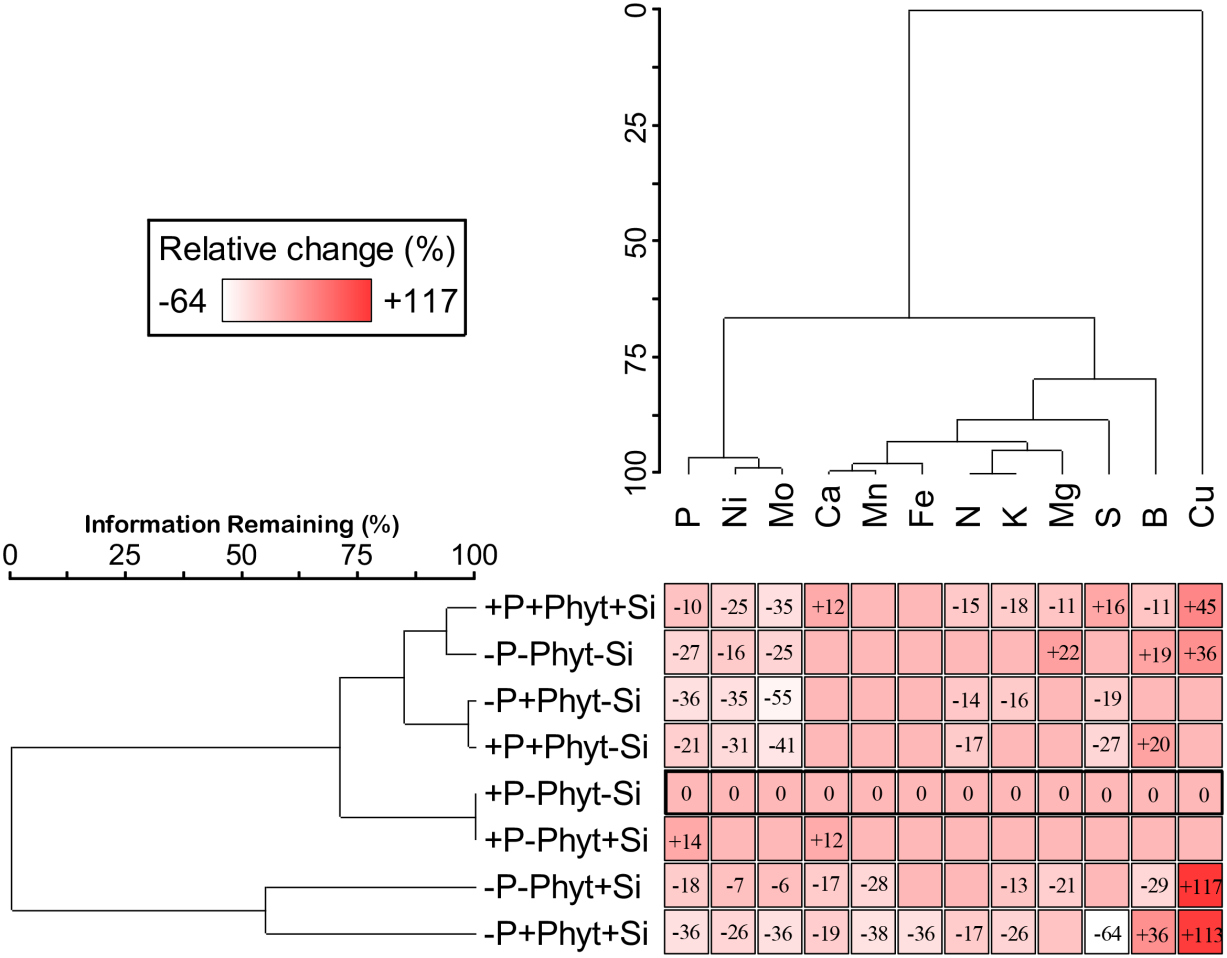
The effect of phosphorus (P) supply, *Phytophthora plurivora* root infection and silicon (Si) addition on the relative change of mineral nutrients concentrations as compared to the control. Two-way cluster analysis presented shows simultaneous groupings of treatments (by their similar effect on nutrient leaf accumulation) and of mineral nutrients (by their similar response to treatment factors). Control (+P–Phyt–Si) is delineated by thick line. Blank cells correspond to nonsignificant variations in leaf nutrient concentration compared to the control. Leaf concentrations of zinc (Zn) are left out because they did not significantly change in any treatment. Data matrix: statistically significant relative change (colour coded: white – highest decrease, dark red – highest increase) of leaf nutrient concentrations compared to the control; Euclidean distance, Ward’s linkage; percent chaining 18%, total sum of squares 37387.6.

**Figure 6:**
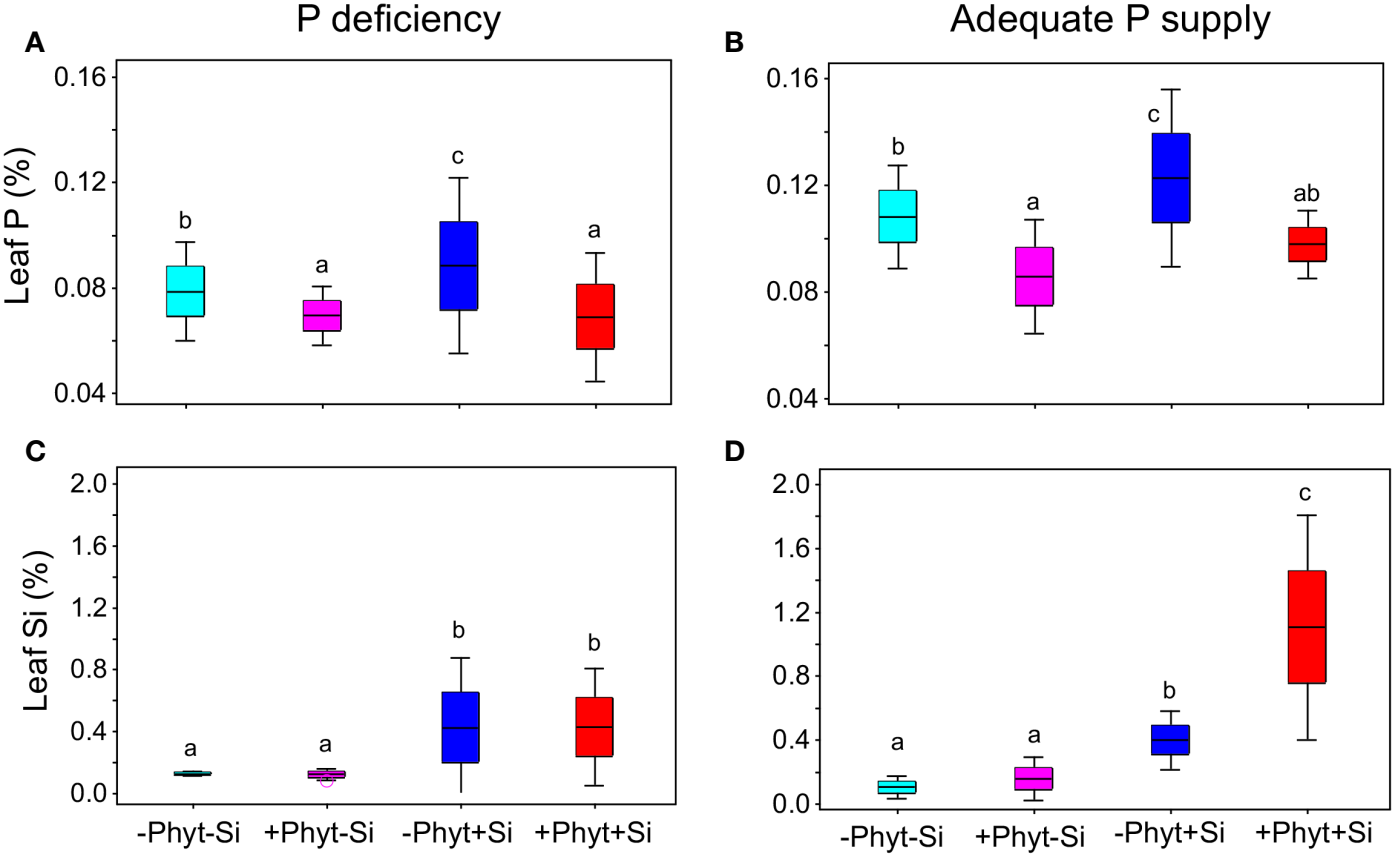
Frequency distribution of leaf P **(A, B)** and Si **(C, D)** concentrations as affected by the addition of Si and *Phytophthora plurivora* root infection at contrasting levels of P supply. Box-plots scaled in standard deviations are shown; whiskers encompass ± 2 S.D. from the mean. P deficiency **(A, C)**; adequate P supply **(B, D)**. Different letters denote significant differences (α=0.05) among the treatment means (10 plants per treatment) for each element detected by Tukey test following each 2-way ANOVA.

**Figure 7:**
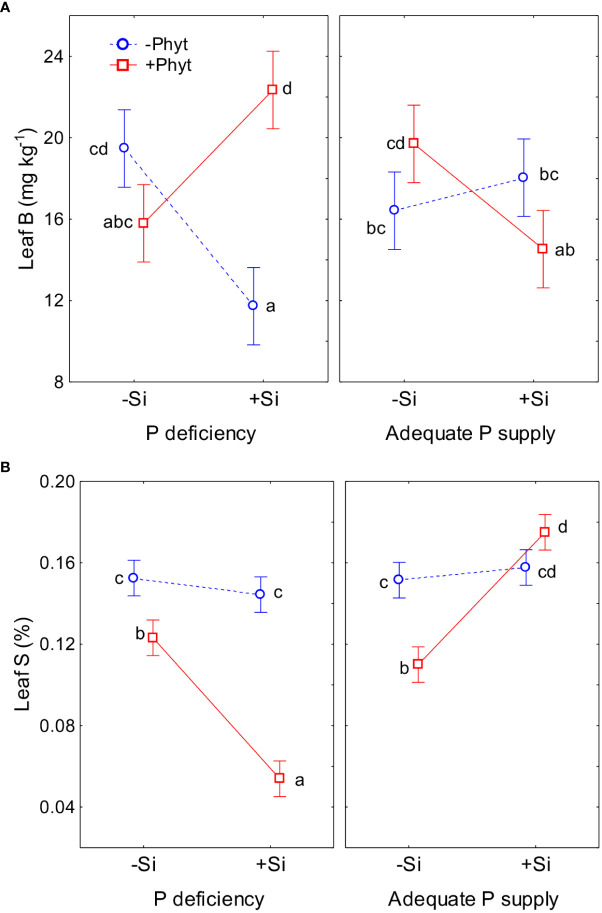
The contrasting effect of silicon (Si) addition on leaf accumulation of boron (B) **(A)** and sulphur (S) **(B)** in 12-week-old pedunculate oak seedlings exposed to different stresses. Statistical interactions phosphorus (P) × *Phytophthora plurivora* (Phyt) × silicon (Si) are shown. B: F(1,72) = 61.1, p<0.0000001, Full model: R^2^adj. 0.50, F(7,72)=12.3, p <0.0000001 **(A)**; S: F(1,72)=93.4, p<0.0000001, Full model: R^2^adj. 0.88, F(7,72)=75.1, p <0.0000001 **(B)**. The same letters indicate no statistically significant differences (α=0.05) among the treatment means (10 plants per treatment) for each element, according to the Tukey test following each 3-way ANOVA. The least square means estimates for the factors are shown. Vertical bars denote 95% confidence intervals.

**Figure 8:**
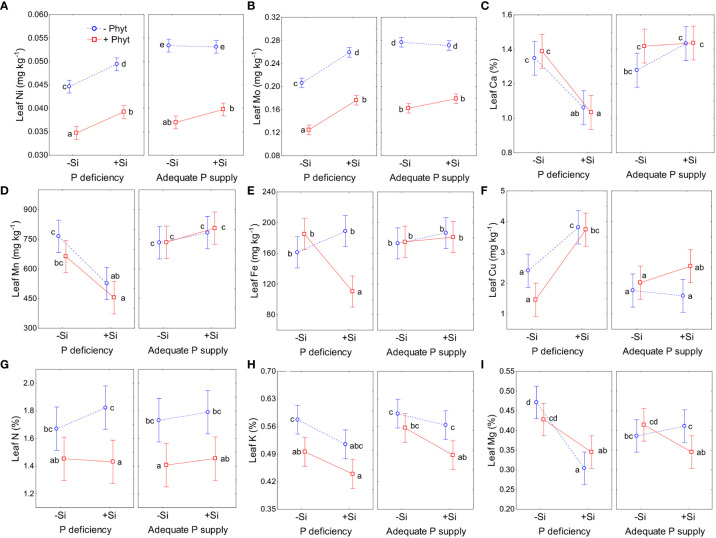
The effect of phosphorus (P) supply, *Phytophthora plurivora* root infection and Si addition on leaf concentrations of the selected nutrients. Statistical interaction phosphorus (P) × *Phytophthora plurivora* (Phyt) × silicon (Si) are shown. Nickel (Ni): F(1,72)=2.78, p=0.10; Full model R^2^adj 0.91, F(7,72)=115.1 **(A)**; Mo: F(1,72)=3.86, p=0.053; Full model R^2^adj 0.94, F(7,72)=179.4 **(B)**; calcium (Ca): F(1,72)=0.23, p=0.63; Full model R^2^adj. 0.48, F(7, 72)=11.5, p<0.0000001) **(C)**; manganese (Mn): F(1, 72)=0.006, p=0.94; Full model R^2^adj. 0.43, F(7,72)=9.6, p<0.0000001 **(D)**; iron (Fe): F(1,72)=11.0, p=0.0014, Full model R^2^adj. 0.33, F(7,72)=6.5, p=0.000006 **(E)**; copper (Cu): F(1,72)=0.48, p=0.84; Full model R^2^adj. 0.48, F(7,72)=11.5, p<0.0000001 **(F)**; nitrogen (N): F(1, 72)=0.54, p=0.47; Full model R^2^adj. 0.26, F(7,72)=5.0, p=0.00012 **(G)**; potassium (K): F(1,72)=0.80, p=0.38; Full model R^2^adj. 0.39, F(7,72)=8.3, p<0.0000001 **(H)**; magnesium (Mg): F(1,72)=9.2, p=0.034; Full model R^2^adj. 0.34, F(7,72) =6.8, p<0.0000001 **(I)**. Different letters denote significant differences (α=0.05) among the treatment means (10 plants per treatment) detected by Tukey test following each 3-way ANOVA. Vertical bars denote 95% confidence intervals.

**Figure 9:**
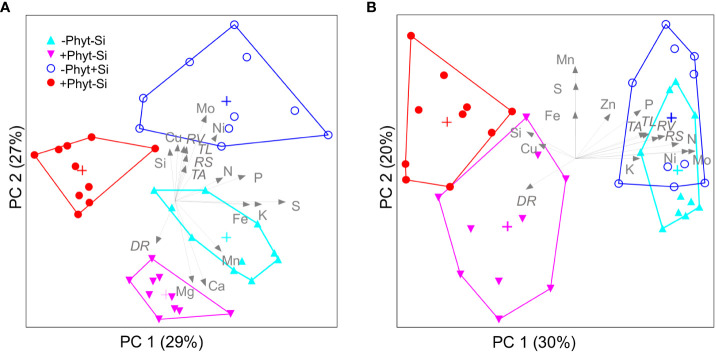
Free ordination (Principal Components Analysis) visualizes the segregation of leaf ionomic profiles caused by *Phytophthora plurivora* root infection and silicon (Si) treatments in young oak seedlings grown at contrasting phosphorus (P) supply. P deficiency **(A)**; adequate P supply **(B)**. Data matrix: leaf concentrations of 14 mineral elements (13 nutrients and Si), 4 treatments, 10 plants per treatment at each level of P supply. The share of the total variance represented by each axis is parenthesized; group centroids (cross) and convex hulls (polygons) are presented. Leaf elemental concentrations, as well as root phenomic parameters which were correlated by more than 25% with the ordination scores, are passively overlaid. The angles and lengths of the radiating lines indicate the direction and strength of relationships of the measured parameters with ordination scores. PC: principal component (significant ordination axis); *RS*: total root surface area; *RV*: total root volume; *TL*: length of thin roots: *TA*: projected area of thin roots; *DR*: percentage of damaged root surface. Triangles: –Si treatments; circles: +Si treatments.

Concomitantly with the changes of plant growth and disease symptoms (**Figures 1**–**4**), the applied treatments (P supply, *P. plurivora* infection, and Si addition) caused complex changes in leaf concentrations of nutrients (**Figures 5**–**9**; **Tables 1**–**3**). No univariate statistically significant changes were detected (by 3-way ANOVA) only for Zn leaf concentrations (mean treatment values ranged from 15 to 18 mg kg^-1^; **Table S7**).

**Table 2:** Differentiation of leaf nutriomic signatures in 12-week-old oak plants in response to phosphorus (P) supply, *Phytophthora plurivora* root infection and silicon (Si) addition.

Treatments	–P–Phyt–Si	–P–Phyt+Si	–P+Phyt–Si	–P+Phyt+Si	+P–Phyt–Si	+P–Phyt+Si	+P+Phyt–Si	+P+Phyt+Si
–P–Phyt–Si	0							
–P–Phyt+Si	45.6	0						
–P+Phyt–Si	38.1	55.3	0					
–P+Phyt+Si	53.2	48.8	51.8	0				
**+P**–**Phyt**–**Si**	**28.8**	**38.1**	**42.1**	**69.3**	0			
+P–Phyt+Si	32.1	43.7	41.8	77.4	**14.8**	0		
+P+Phyt–Si	22.8	24.2	53.8	45.1	**38.2**	38.2	0	
+P+Phyt+Si	33.6	45.3	33.6	50.1	**41.1**	41.0	25.9	0

The relative differences among the nutriomes are expressed as Gower’s distance (in %). All the differences were found to be statistically significant by the MRPP test (at α=0.05). Data matrix: leaf concentrations of 13 nutrients (averages of 10 plants per treatment), 8 treatments. Light grey: P deficient treatments; Dark grey: adequate P supply. The values referring to the control (adequate P supply, no pathogen, no Si) are bolded.

**Table 3:** The relative effect strength of the treatment factors (*Phytophthora plurivora* root infection and Si addition) on segregation of leaf nutriomic profiles of oak seedlings grown at contrasting phosphorus (P) supply levels.

Treatment factors	P deficiency		Adequate P supply	
	Average F	p	Average F	p
*P. plurivora* infection	66.8	0.0002	93.0	0.0002
Si addition	32.0	0.0002	8.7	0.0002
*P. plurivora* × Si	10.3	0.0002	6.7	0.0004

Average F values and their associated p values of the SumF analyses are shown. Data matrix: leaf concentrations of 13 nutrients, 4 treatments, 10 plants per treatment, at each level of P supply.

The pairwise comparisons of leaf nutriomic profiles as fingerprints of mineral element concentrations showed, however, that no two nutriomes, of the eight groups compared, were statistically the same (MRPP analysis; **Table S8**), and the relative multivariate difference among the ionomes ranged from about 15% to about 77% (**Table 2**). Overall, compared to the control (+P–Phyt–Si), the strongest ionome change (by about 70%) was induced by the combination of both stresses and no Si amendments (–P+Phyt–Si). The smallest differences were observed between a) +/– Si to the unstressed seedlings, and b) healthy P-deficient plants, and adequately P-fed ones infected by *P. plurivora* when no Si was added. The conspicuous changes of leaf nutrient concentrations induced by the addition of Si (compared to –Si treatments) under the three stresses were the strongest under the combined stress (about 52%), while under P deficiency and *P. plurivora* root infection this relative difference in nutriome signatures was about 46% and 26% respectively (**Table 2**). In the same line, Si addition had a stronger effect (i.e., more of the observed variance “explained” by the application of Si) on segregation of leaf nutriomes under P deficiency than under adequate P supply (**Table 3**). The average F value (statistically, signal-to-noise ratio) for the factor Si addition in the SumF analysis was about 3.6-fold higher under P deprivation compared to the adequate P supply, what comprises considerably stronger effect of Si on the changes of leaf nutrient concentrations under the more severe stress.

The change of nutriomes relative to the control and a concomitant grouping of treatments and of nutrients is shown in **Figure 5**. The significant change in leaf nutrient concentrations (7 treatments and 13 mineral nutrients) compared to the control occurred in 55% of the cases, and this relative change ranged from –64% for S to +117% for Cu. The presented results illustrate a very complexly orchestrated “symphony” of the plant physiological responses at the level of leaf nutrient accumulation to the presence of a biotic, of an abiotic, and of Si (and to their combinations). Firstly, the strongest change in leaf nutrient signatures (relative change and number of elements affected) was induced by Si addition to the seedlings exposed to both stresses (–P+Phyt+Si) simultaneously. Next, the application of Si under stress(es) did not revert the nutriomic signatures to the level of the control. Finally, the addition of Si under each stress caused a stress-specific response in nutrient uptake and accumulation (**Table 4**). Thus, the Si-induced change in the leaf nutriome of oak seedlings exposed to both P deficiency and root infection was not a simple sum of responses to each stress individually. The details of nutrient accumulation change in response to the treatments are further shown in **Figures 6**–**8**.

**Table 4:** The effect of silicon (Si) addition on the change of leaf nutrient accumulation under stresses.

Stress	Effect of Si on leaf nutrient concentrations under stress	
	Increase	Decrease
P deficiency	Cu (60%), P (18%), Mo (26%), and Ni (9%)	B (67%), Mg (35%), Mn (31%), and Ca (21%)
*P. plurivora* root infection	S (60%), P (17%), Mo (10%), and Ni (8%)	B (26%), Mg (15%), and K (12%)
P deficiency + *P. plurivora*	Cu (256%), B (41%), Mo (18%), and Ni (11%)	S (55%), Fe (40%), Mn (31%), Ca (26%), Mg (19%), and K (12%)

Statistically significant changes (t-test, α=0.05) in elemental concentrations induced by Si application are presented; the percentage change (relative to the stress treatment without Si) are given in parentheses.

The leaf accumulation of P was responsive to all the treatments (**Figure 5**). The lack of P and the root infection by *P. plurivora* decreased leaf P levels. At low P supply, the addition of Si to the non-infected plants increased leaf P concentrations by about 17% (**Figure 6A**). At an adequate P supply, Si treatment improved leaf P status in the non-infected plants comparably (about 14%), but it also recuperated the leaf P concentrations of the infected plants to the level of the control (**Figure 6B**), what was not observed under P deficiency.

The grouping of nutrients according to their similar response to the treatments singled out Cu, B, and S (**Figure 5**). The clearly contrasting patterns of leaf accumulation affected by Si supply were observed for B and S at different levels of P availability (**Figure 7**). Leaf B was decreased when Si was applied to each stress individually, but strongly increased with Si under the combined stress (**Figure 7A**). Root infection decreased leaf S status irrespectively of P supply; the application of Si to the infected seedlings however strongly suppressed S accumulation under P deficiency, but stimulated it at adequate P supply (**Figure 7B**). For the other nutrients, the effect of Si was overall stronger under P deficiency then at an adequate P supply (**Figure 8**; see also **Table 3**). Furthermore, three groups of nutrients responded similarly to the experimental treatments (clustering of nutrients in **Figure 5**). In the first group, P (**Figures 6A, B**) and in particular Ni (**Figure 8A**) and Mo (**Figure 8B**) concentrations were consistently decreased by the applied stresses and increased by Si. For Ni and Mo, t-test confirmed significant differences between +P+Phyt–Si and +P+Phyt+Si treatments, which were not detected by the ANOVA full model. For the second group (Ca and Mn, subsequently joined by Fe) the application of Si changed the accumulation pattern only under P deficiency: Ca (**Figure 8C**) and Mn (**Figure 8D**) decreased when Si was added to P-deficient seedlings, irrespectively of the presence of the pathogen, while Fe (**Figure 8E**) was decreased only when Si was supplied to the plants under the combined stress. Leaf Cu (**Figure 8F**) on the other hand showed a unique response (see also **Figure 5**), but contrasting to Ca and Mn: it was increased by Si addition to the seedlings exposed to P deficiency, both the healthy and the infected ones. In the third group (N and K, subsequently joined by Mg; **Figure 5**), leaf concentrations of N (**Figure 8G**) were not affected by Si (only the presence of the pathogen was a significant predictor), while the seedlings supplied with Si had on average 5.4% lower K (**Figure 8H**) concentrations. Leaf Mg (**Figure 8I**) was clearly and consistently decreased by Si application under all three types of stress.

Finally, in P-deficient oak seedlings the addition of Si led to a 3-fold increase in leaf Si concentration, irrespectively of the pathogen presence (**Figure 6C**). Under an adequate P supply, on the other hand, Si treatment caused a similar increase in leaf Si in the non-infected plants (about 3-fold), while in the infected plants this increase was about 7-fold (**Figure 6D**). Thus, when adequate supply of nutrients and Si was provided, the presence of biotic stress increased leaf accumulation of Si 2.75 times compared to the pathogen-free conditions.

Free ordination of leaf elemental profiles (**Figure 9**) also summarizes covariations of all the measured oak parameters as affected by the experimental treatments. Of the total encountered variation in leaf mineral concentrations, two significant PCA axes represented 56% in plants grown under P deficiency (**Figure 9A**), while 3 axes (i.e. 3 principal components) captured 64% at adequate P supply (**Figure 9B**); for the clarity, only the first two ordination axes are shown. At both levels of P supply, the first principal component chiefly separated the element signatures of the non-infected from the infected plants. At an adequate P supply, infected plants were clearly separated by lower concentrations of N, P, K, Zn, Ni, and Mo from the healthy ones (**Figure 9B**), while under P deficiency the PC 1 clearly distinguished only +P+Phyt treatment (**Figure 9A**). Furthermore, at both levels of P supply the PC 2 captures the differences in leaf nutrient concentrations caused by the application of Si. At low P supply (**Figure 9A**), the addition of Si strongly decreased Mg and Ca, while at adequate P supply (**Figure 9B**) increased S and Mn. At an adequate P supply, leaf Si was higher when Si was added in the presence of the pathogen compared to healthy plants (**Figure 9B**). The measured root phenes were correlated with the second ordination axis (PC 2, which reflects the effects of +/– Si addition) under P deficiency (**Figure 9A**), and with PC 1 (which reflects the effect of +/– root infection) at adequate P supply (**Figure 9B**). Consistently, plant dry biomass showed a stronger correlation with the changes in nutriome under P deficiency (Pearson r^2^ with PC 2 of 24%) compared to an adequate P supply (r^2^ with PC 1 of 15%).

## 4 Discussion

### 4.1 Overall effect of Si supply

This work demonstrates the beneficial effect of Si addition on the growth parameters of pedunculate oak seedlings when subjected to both P deficiency and *P. plurivora* root infection. It also provides an insight into a complex nutrient crosstalk mediated by stress and Si supply, which influence acquisition and leaf accumulation of nutrients. Silicon-mediated changes of the nutrient profiles were obviously a primary physiological adaptation, and these changes were clearly detectable in all of the 8 treatments (**Figures 5**–**9**; **Tables 2**–**4**; see also **Table S8**). The ameliorative effect of Si on the root architecture traits (**Figure 4**) could only be detected in the presence of biotic stress, while the Si-induced improvement of biomass accumulation (**Figure 2**), and of root damage caused by the pathogen (**Figure 3**) were established clearly only in the combination of stresses. Recent studies found complex changes in ionome profile but no response of biomass accumulation of two herbaceous crops after 10 days of macronutrient deficiencies (Courbet et al., 2021), and 22 days of micronutrient deficiencies (D’Oria et al., 2021). It should be also noted that detection of “statistically significant” signals (or the lack of it, e.g., considering the ameliorating effect of Si on plant growth and root damage at an adequate P supply; see **Figures 2**, **3**) in young wild plants is commonly hampered by their intrinsically high growth variability, contrary to working with commercial crop cultivars and despite our efforts to select as uniform experimental plants as possible. The lack of statistical significance in nutrient accumulation patterns when the p value of the conservative Tukey’s test was very close to 0.05 should thus also be cautiously interpreted in the cases where other analysis (ordination) indicated some conspicuous trends. Furthermore, it is evident that the alterations in leaf micronutrient concentrations cannot be attributed to the concentration effect resulting from reduced growth. This is apparent, *inter alia*, when considering the contrasting responses of elements such as Cu and Mn under stress conditions (**Figure 8**).

Pedunculate oak has a relatively high concentrations of Si in leaves, i.e., in average 1.17% and up to 3.04% in the mature trees (Lisztes-Szabó et al., 2019), which is in the order of magnitude of Si-accumulating species (Ma et al., 2023). It has been proposed that, under stress conditions, species with intrinsically higher Si accumulation tend to have a stronger reaction to Si supply (Coskun et al., 2019). For example, Si had a stronger effect on transcriptome and overall symptoms in Si accumulating wheat compared to non-accumulating Arabidopsis with powdery mildew (Fauteux et al., 2006; Chain et al., 2009). Low availability of P can increase Si shoot concentrations (provided that Si availability in soil is sufficient) in Si-accumulating gramineous species (Chaiwong et al., 2018; Minden et al., 2020). In the same line, P deficiency was found to trigger increased Si leaf concentrations in Si-accumulating wheat, but not in non-accumulating oilseed rape (Courbet et al., 2021). Here, we did not find that Si leaf accumulation in +Si treatments of the non-infected plants was increased at low P compared to adequate P supply, but in the +P oak seedlings we did detect about 3-fold higher Si levels in the infected compared to the non-infected ones (**Figures 6C, D**). Such increased uptake and leaf accumulation of Si has previously been reported in plants attacked by herbivores (Massey et al., 2007) and infected by fungal pathogen (Gao et al., 2011).

It has been suggested that the ameliorative effect of Si chiefly occurs under some stress (Coskun et al., 2019). On the other hand, severe deprivation of Si (in hydroponic experiments) can induce very complex changes as an increase of Ca, Mg, K, S, Mn and Zn in wheat, and a decrease in N and Mo in oilseed rape (D’Oria et al., 2021). Our work further demonstrated that the stronger the stress, the clearer was the response to Si addition, though even in –Si treatments the available soil Si (about 40 mg kg^-1^) was far beyond its deficiency threshold (Liang et al., 2015). The application of Si to oak seedlings under the most severe stresses (P deficiency + *P. plurivora* root infection) resulted in the strongest improvement of biomass accumulation (**Figure 2**), root damage (**Figure 3**) and visual symptoms (**Figure 1**), and also had the strongest statistical signal of altering leaf nutriome profiles (**Tables 2** and **3**) affecting thus leaf accumulation pattern of 11 out of 13 nutrients (**Table 4**; **Figure 5**). It has previously been suggested that the beneficial effect of Si on plant disease might be more prominent under abiotic stress than under non-stressed conditions (Reynolds et al., 2009). Interestingly, we further found that leaf concentrations of micronutrients Ni and Mo were highly responsive (actually more clearly responsive than P) to all the three stresses (consistent decrease) and to the application of Si under these stresses (consistent increase; see **Figure 5** and **Table 4**), having also a very narrow range of within-treatment variation (**Figures 8A, B**; coefficient of variation mostly about 5%). This indicates that leaf Ni or Mo concentrations might be considered a potential proxy for nutriome changes induced by Si application under stress conditions.

### 4.2 Responses to P deficiency

So far, the response of leaf ionomics to P deficiency has not been unequivocally established (except for decrease in P concentrations). In addition, very little is known on molecular determinants of alterations in ionome caused by P supply fluctuations (Wang et al., 2020). For instance, Baxter et al. (2008) found that low P caused an increase of B, Fe, and Zn, and a decrease of Co and Cu leaf concentrations in Arabidopsis. Wang et al. (2020) likewise reported that P starvation resulted in an increase of B, Fe, S, and Zn, and a concomitant decrease of Ca, Cu, K, and Mg in oilseed rape. On the contrary, Courbet et al. (2021) observed a P-deficiency caused increase in the uptake of Si in wheat, and a decrease in the uptake of majority of the nutrients in both oilseed rape and wheat. Comparing P deficiency to full NPK fertilization treatment, Watanabe et al. (2022) found some similarities in response of four crops (wheat, maize, sunflower, and soybean): decrease of Ca and K, and increased accumulation of Cu. In tea plants, P deficiency decreased leaf S, and a concomitantly increased Cu, Zn, Mn, and Fe (Ding et al., 2017), while in the leaves of sour pummelo it led to the increased K, S and B and decreased N, Mn and Cu concentrations (Meng et al., 2021). On the other hand, Ji et al. (2019) did characterize the differences in ionomes of the two adult subtropical *Quercus variabilis* populations growing on soils with contrasting P availability, but these soils also had different availability of other nutrients, so that the sole effect of P supply could not be concluded. In the present study, we showed that P deficiency *per se* in the non-infected oak seedlings (–P–Phyt–Si) caused about 29% difference in leaf nutriome compared to the control (+P–Phyt–Si, **Table 2**), which was clearly reflected in a decrease in P, Ni, and Mo, and a simultaneous increase in Cu, B, and Mg leaf concentrations (**Figure 5**). This change in nutrient uptake and accumulation induced by suboptimal P supply was concomitant with a comparable decrease in biomass accumulation (by about 23%, **Figure 2**), but also with decreased total root volume by about 27% and projected area of thin roots by about 20% (**Figures 4B, D**).

The addition of Si strongly altered the ionomic fingerprint of non-infected P-deficient 12-week-old pedunculate oak seedlings (**Table 4**; see also **Table 2**) and eliminated the leaf visual symptoms of P deficiency (**Figure 1**). During the course of our experiment, however, the addition of Si was not detected to affect plant dry biomass (**Figure 2**) nor the root phenes (**Figure 4**). Furthermore, we could not detect the effect of P deficiency *per se* on root growth stimulation in oak seedlings (**Figure 4**). This might be due to the young growth stage of the plants; for instance, Newnham and Carlisle (1969), working with comparably young (85 days) oak seedlings could as well not yet have discerned any effect of P deficiency on stimulated root growth and root to shoot ratio. In our study, due to an extremely poor condition of the seedlings exposed simultaneously to both stresses (without Si addition, see **Figure 1**), it was not possible to further extend the experiment.

The supply of Si to the non-infected seedlings grown under the lack of P (–P–Phyt–Si *versus* –P–Phyt+Si treatments) caused a strong relative change of leaf nutriome by about 46% (**Table 2**), clearly manifested in a further increase of Cu (1.7-fold, **Figure 8F**) and improved leaf P (by 17%, **Figure 6A**), and a prominent decrease (by about 70%) in B (**Figure 7A**); the leaf concentrations of Ca, Mg, and Mn were also decreased in +Si treatments (**Table 4**; see also **Figures 5**, **8**, **9A**). So far, data on the effect of Si supply on nutrient accumulation under P deficiency is extremely scarce. In tomato, a Si non-accumulating species with not functional Lsi2 efflux transporter for xylem loading with Si (Sun et al., 2020), Zhang et al. (2019) reported Si-induced increase in P, Mg, Fe, and Cu, and concomitant decrease in Zn leaf concentrations. The alleviating effect of Si on plant growth under P limiting conditions has been reported so far for five annual food crops (review in Pavlovic et al., 2021); however, the precise nature of this effect remains unclear. Kostic et al. (2017) showed that Si supply to P deficient wheat plants improved their growth, and increased the concentration of shoot P to the level of P-fertilized plants through the up-regulation of the expression levels of *TaPHT1;1* and *TaPHT1;2* involved in inorganic P uptake, as well as through an increased root exudation of malate and citrate which can improve P availability in the rhizosphere. Accordingly, the addition of Si to the healthy P deficient oak seedlings in the present study increased leaf P concentrations, though not quite to the level of +P plants (**Figures 6A, B**). Silicon fertilization increased leaf P concentrations also in P-fertilized trials what might imply that the rate of P addition in our experiment still has not enabled luxury consumption of this nutrient. This is essentially in accordance with findings on wheat under mild P limitation, as reported by Neu et al. (2017), providing further evidence that Si supply may enhance the optimal level of P supplementation (Ma and Takahashi, 1990). This Si-mediated improvement of leaf P status could have further led to some complex changes in the uptake and accumulation of other nutrients. For instance, Wang et al. (2018) showed that P-deficiency stimulated increased B uptake in dicots (which have intrinsically higher B requirements), but not in monocots, what is in accordance with our findings in pedunculate oak (**Figures 6A**, **7A**). In both rhizobox and hydroponic experiments, these authors demonstrated the up-regulation of BnBOR1 transporter in oilseed rape by the lack of P. We further showed that when plant P status was improved by Si addition (**Figure 6A**), the uptake and leaf accumulation of B was decreased (**Table 4**; **Figure 7A**). Finally, Si addition to P-deficient seedlings did not revert the complete analysed leaf nutriomic signature to the one in the control plants (+P–Phyt–Si; **Figure 5**). Following Si application, leaf Cu concentrations were about 1.2-fold higher, while the levels of P, K, Ca, Mg, Mn and B were lower compared to the control.

### 4.3 Responses to *P. plurivora* root infection

The ameliorative effect of Si on the consequences of fungal and bacterial diseases on different crops has been summarized in more than 100 publications (Wang et al., 2017; Coskun et al., 2019; Debona et al., 2023); majority of the published works dealt with changes on the levels of metabolics production and gene expression, while the effects on ionomes are extremely rarely studied. For instance, the positive effect of Si on plants infected by the pathogenic genus *Phytopthora* was reported in bell pepper (*P. capsici*, French-Monar et al., 2010), cucumber (*P. melonis*, Mohaghegh et al., 2011), and soybean (*P. sojae*, Guérin et al., 2014; Rasoolizadeh et al., 2018), but the leaf nutrient concentrations were not analysed in these studies.

In the present study, root infection by *P. plurivora per se* (comparing +Phyt *versus* –Phyt treatments, at adequate P supply and no addition of Si) caused a decrease in total biomass production by about 17% (**Figure 2**), more than 3 times higher incidence of necrotic lesions on roots (**Figure 3**), prominent decrease in root morphological parameters (by 40-57%, **Figure 4**), and a concomitant relative change of the leaf ionome by about 38% (**Table 2**). This pathogen-induced modification of nutrient uptake and accumulation was clearly reflected in the increased leaf concentrations of B, and simultaneous decrease in the concentrations of P, S, N, Ni, and Mo (**Figure 5**). Leaf Ca also tended to increase (by about 11%, **Figure 8C**) but the signal was not significant by the t-test. In comparison, de la Fuente et al. (2013) found (out of 12 elements analysed) significant increase of Ca and decrease of P in tobacco plants infected by xylem parasitic bacterium *Xylella fastidiosa*. *Phytophthora*-induced decrease of leaf S was observed in avocado (Whiley et al., 1987). Nicolas et al. (2019), using nutrient balance approach, observed a decrease of P relative to the concentrations of N and S in lettuce infected by the bacterium *Xanthomonas campestris*. In rice infected by blast fungus (*Magnaporthe oryzae*), leaf concentrations of the measured nutrients (Ca, K, Mg and Fe) were increased (Gao et al., 2011). In general, increased concentrations of Ca, which acts as secondary messenger, are observed as a part of plant immune response to pathogens (Yuan et al., 2017). The damage and finally the necrosis of root tissue by this hemibiotrophic oomycete disrupts nutrient acquisition and uptake, ultimately decreasing leaf nutrient (primarily P and N) status and decreases photosynthesis, leading to the accumulation of carbohydrates in leaves (Whiley et al., 1987; Maurel et al., 2001; Sghaier-Hammami et al., 2013), So, both stresses, P deficiency and root disease, caused similar growth decrease (**Figure 2**), purple leaf coloration (**Figure 1**), lower P (**Figures 6A, B**), and higher B accumulation relative to the control (**Figures 5**, **7A**). However, pathogenic microbes have much more complex relation with host plant for nutrients. For instance, Yuan et al. (2010) showed that the effectors of xylem-inhabiting bacterium *Xanthomonas oryzae* can transcriptionally activate Cu transporter genes (*COPT1* and *COPT5*) in the host plant and thus remove Cu (which has strong antimicrobial properties) from xylem, facilitating the *in planta* spread of the pathogen.

The supply of Si to the infected oak seedings conspicuously modified the pattern of uptake and leaf accumulation of nutrients. Compared to –Si treatments, addition of Si led to a lower concentration of B (by about 40%), Mg, and K, while the levels of S (by about 55%), P, Ni, and Mo were elevated (**Table 4**; see also **Figures 7**, **8**), comprising a relative difference of the respective ionomes of about 26% (**Table 2**). The leaf concentrations of Ca were not affected by Si supplementation. Concomitantly, all the four measured root traits were improved by Si addition (by 32-63%, **Figure 4**), though the values of healthy plants were not achieved. In particular, the length and the projected surface area of thin roots were increased by 63% and 56%, respectively, when Si was added (**Figures 4C, D**). Thin roots are an important indicator of overall oak vitality (McConnell and Balci, 2015; Mosca et al., 2017). Visual symptoms of disease on leaves disappeared when Si was added (**Figure 1**), and pathogen-induced damage of the root tended to be lower (**Figure 3**).

### 4.4 Responses to combined stress

Simultaneous exposure to P deficiency and *Phytopthora plurivora* root infection was a very severe stress for 12-week-old seedlings (see visual symptoms, **Figure 1**); it caused the most intense growth suppression of about 34% (relative to the control, **Figure 2**), and about 7 times more root damage (**Figure 3**), while the decrease in root phenes (by 1.8 to 2.7 times) was comparable to the effect of pathogen infection alone (**Figure 4**). Compared to the control, the leaf nutriomic profile of the seedlings exposed to both stresses simultaneously comprised a relative difference of about 42%, more than the change caused by each stress individually (**Table 2**). The concentrations of P, S, N, Ni, and Mo were decreased under the combined stress (similar to the response of the biotic stress alone), but also K was decreased (**Figure 5**). We noticed just a trend of leaf Mg increase (by 11%) but not significant (t-test p=0.53). The supply of Si (relative to –Si treatment) to the seedlings simultaneously exposed to the two stresses prominently relieved the symptoms on leaves and roots (**Figure 1**), improved the growth (DW per plant increase by 18%, **Figure 2**), and decreased the extent (about 2 times) of necrotic lesions on roots caused by the pathogen (**Figure 3**). Concomitantly, Si supply strongly altered the leaf nutrient signature (relative difference to –Si plants about 52%, higher than under each stress individually; **Table 2**), significantly increasing accumulation of Cu, B, Mo, and Ni, and decreasing S, Fe, Mn, Ca, Mg, and K concentrations (**Table 4**; see also **Figures 5**, **7**, **8**, **9A**).

### 4.5 Outlook: Si and nutrient crosstalks

The ameliorative effect of Si generally tends to countermand the effect of stress (reviewed by Coskun et al., 2019). This has been clearly demonstrated for biotic stress at the gene expression level. Overall transcriptomic response of plants infected by fungal pathogens and treated with Si was the same as the non-infected control (Fauteux et al., 2006; Chain et al., 2009; Rasoolizadeh et al., 2018). Effects of biotic stress on leaf nutrient profiles are seldom reported; Gao et al. (2011) showed that Si reversed the rice blast-induced changes of nutrients (Ca, K, Mg, and Fe) to control levels. For abiotic stresses, on the other hand, the evidence of reversal is ample on the physiological/biochemical level, but very scarce on the level of leaf nutrient profile. In the present study we did not find any parameter to be brought back to the control level by Si. Nevertheless, Si application did result in a significant trend of reversal of stress-induced changes in growth (**Figures 1**–**4**) towards the control. At the level of nutriome, furthermore, the supply of Si tended to abolish the changes (relative to the control) of P, B, Mg, Ni and Mo (but not Cu, which was even further strongly increased) under P deficiency; and P, B, S, Ni and Mo (but not N, which was unaffected by Si) under pathogen attack (**Figure 5**; **Table 4**; see also **Figures 6**–**8**). Interestingly, under the combined stress Si addition caused very complex changes (**Figure 5**; **Table 4**), but reversal trends in leaf nutrient accumulation were observed only for Ni and Mo (**Figures 8A, B**). The supply of Si to the oak seedlings exposed simultaneously to P deficiency and *P. plurivora* did not improve leaf P status (**Figure 6A**), but did improve overall growth and appearance (**Figures 1**–**4**), concomitantly profoundly altering leaf status of 11 out of 12 nutrients that showed any change with the treatments applied (**Figure 5**). Finally, the most interesting finding is that Si addition affected also leaf concentrations of elements which had not been influenced by the respective stress itself. In particular, Si also decreased Ca and Mn (which were not affected by P deficiency), K and Mg (which were not affected by the infection), and led to increase in Cu and B and decrease in Ca, Mg, Fe, and Mn, which were not affected by the combined stress (**Table 4**; **Figures 5**, **7**, **8**). Thus, our work clearly demonstrated that Si addition under stress affects the uptake and accumulation of a higher number of nutrients than does the respective stress alone.

In general, our results support the notion that physiological responses of plants in combined stresses are unique and cannot be directly extrapolated from the results of individual stresses (reviewed for heat, drought, light intensity and salinity by Mittler and Blumwald, 2010). Moreover, the mechanism underlying the ameliorative effect of Si on stressed plants has so far remained unknown; no biochemical reactions and gene regulation pathways directly triggered by Si have been identified. Currently, apoplast is considered the main place of Si actions. Apoplast obstruction hypothesis has been proposed to explain the effect of Si on relieving metal toxicity stress (Coskun et al., 2019), while Rasoolizadeh et al. (2018) linked Si to apoplastic effector/receptor interactions under *Phytopthora sojae* infection; for nutrient deficiencies nonetheless, no model has yet been proposed. In fact, we can not explain the Si-mediated changes of the majority of the examined nutrients under the three stresses. For instance, leaf B (**Figure 7A**) was also found to increase in different species under P deficiency (Baxter et al., 2008; Wang et al., 2020; Meng et al., 2021), which was further confirmed by increased expression of B transporters (Wang et al., 2018). In our work, the addition of Si under individual stress conditions tended to restore B but also P accumulation levels closer to the control. However, when the two stresses were combined, Si supply did not improve the leaf P status; yet, in the presence of Si, the accumulation of B (which plays a role in phenolics and lignin synthesis as a response to biotic stress; Weinmann et al., 2023) increased by 37%. Furthermore, the activation of primary S metabolism, including increased uptake of S and a subsequent increased production of S-containing defence compounds (“S-enhanced defence”), independent of external S supply and mediated by jasmonic acid signal, have been firstly described in Arabidopsis/*Alternaria brassicicola* pathosystem (Kruse et al., 2007). However, S uptake in Arabidopsis can also be increased by P deprivation (Misson et al., 2005), possibly, among other unknown factors, due to P-deficiency induced increased synthesis of sulpholipides (Ticconi and Abel, 2004). Nevertheless, in the combined stress of P deficiency and pathogen, we found a strong decrease of S leaf status when Si was added (**Figure 7B**), yet together with the clearly improved growth variables (**Figures 1**–**4**).

The present pioneering work only initiated the understanding of the complexity of stress- and Si-mediated nutrient crosstalks. From a large number of ionome studies without intended stress it became clear that elements in an ionome do not change independently; some of the relations among nutrients are known to some extent, while many are yet to be discerned (Baxter, 2015). While some elemental crosstalks induced by nutrient deficiency have already been reported, recently also Si has been included (P-Fe-Si crosstalks, Chaiwong et al., 2018; Chaiwong et al., 2020). Moreover, even a single nutrient stress causes multiple metabolic disturbances (Maillard et al., 2016); they are chiefly mediated by hormone signalling, primarily jasmonic acid (Fauteux et al., 2006; Chain et al., 2009; Aparicio-Fabre et al., 2013; Khan et al., 2016). The co-ordinated plant responses under multiple stresses become even more complex. For instance, P deficiency responses might, in the first week of stress, via enhancing jasmonate signalling, increase the resistance of Arabidopsis, tomato and tobacco to a moth larva (Khan et al., 2016). On the contrary, in a longer experiment with Arabidopsis and synthetic bacterial community Castrillo et al. (2017) showed that under P deficiency PHR1 acts as a master transcriptional factor which strongly promotes jasmonate accumulation, enhances phosphate starvation response, but represses plant immune response to the microbes competing for P.

## 5 Conclusions

As far as we are aware this is the first report on the effect of Si on modulating plant responses under an abiotic and a biotic stress. The application of Si had the strongest ameliorative effect on growth, root health and root phenome of oak seedlings under the most severe stress conditions (combination of P deficiency and *P. plurivora* root infection), where it differentially affected the uptake and leaf accumulation in 11 out of 13 analysed nutrients. Silicon tended to reverse the pattern of change of some, but not all, leaf nutrients affected by stresses: P, B and Mg under P deficiency, and P, B and S under pathogen attack, but also Ni and Mo under all three stresses. Also, Si affected some nutrients that were not changed by a particular stress itself, and decreased leaf Mg levels under all the stresses. This exploratory work presents the complexity of nutrient crosstalk under three stresses and opens more questions about genetic networks that control plant physiological responses. Practically, we show a potential of Si application to improve P status and root health in oak seedlings, particularly in nurseries.

## Data availability statement

The original contributions presented in the study are included in the article/**Supplementary Material**. Further inquiries can be directed to the corresponding authors.

## Author contributions

IK: Conceptualization, Data curation, Formal Analysis, Investigation, Methodology, Writing – original draft. NN: Data curation, Investigation, Methodology, Software, Validation, Visualization, Writing – original draft, Writing – review & editing. SM: Data curation, Methodology, Software, Writing – review & editing. IM: Data curation, Software, Validation, Writing – review & editing. JP: Data curation, Formal Analysis, Validation, Writing – review & editing. AP: Data curation, Formal Analysis, Writing – review & editing. MN: Conceptualization, Funding acquisition, Methodology, Resources, Supervision, Writing – review & editing.
