# Supplementary material for: Silicon modifies leaf nutriome and improves growth of oak seedlings exposed to phosphorus deficiency and Phytophthora plurivora infection
Source: Front Plant Sci. 2023 Aug 29;14:1265782. doi: 10.3389/fpls.2023.1265782 (PMC10495579; doi:10.3389/fpls.2023.1265782)
Supplement: Supplementary file 1 [file DataSheet_1.pdf]

Supplementary Material

Table S1. The impact of experimental factors (P supply, *Phytophthora plurivora* infection and Si addition) on dry weight of 12-weeks old oak plants (mg plant^{-1}). The significant coefficients of the 3-way ANOVA model (80 samples; α=0.05) are shown. Full model: R^2adj. 0.47; model F=10.7, p <0.0000001.

Parameter	Plant DW (mg)	t value	-95% CL	+95% CL
Overall mean	2871.8	68.0	2787.7	2956.2
P deficiency	−269.2	−6.4	−353.4	−185.1
Phytophthora infection	−217.5	−5.1	−301.7	−133.3
Si addition	85.4	2.6	22.3	169.6

CL-confidence limit of the coefficient estimate.

Table S2. The impact of experimental factors (P supply, *Phytophthora plurivora* infection, and Si addition) on the visually estimated root damage (percentage of root surface with the observed necrotic lesions). The significant coefficients of the 3-way ANOVA model (80 samples; α=0.05) are shown. Full model: R^2adj. 0.71, F(7,72)=28.5, p<0.0000001.

Parameter	Damaged roots (%)	t value	-95% CL	+95% CL
Overall mean	26.7	18.5	23.8	29.7
P. plurivora infection	16.4	11.4	13.6	19.3
Si addition	-6.2	-4.3	-9.1	-3.3
P deficiency	5.1	3.5	2.2	7.9
P. plurivora × Si addition	–5.9	–4.1	–8.8	–3.1
P deficiency × *P. plurivora*	5.6	3.9	2.7	8.4
P deficiency × *P. plurivora* × Si addition	-3.3	-2.3	-6.2	-0.4

CL-confidence limit of the coefficient estimate

Table S3. The impact of experimental factors (P supply, *Phytophthora plurivora* infection, and Si addition) on the total root surface area per plant (cm^2). The significant coefficients of the 3-way ANOVA model (80 samples; α=0.05) are shown. Full model R^2adj. 0.73, F(7,72)=32.1, p<0.0000001.

Parameter	Projected root surface (cm^2 per plant)	t value	-95% CL	+95% CL
Overall mean	116.4	76.9	113.3	119.4
P. plurivora infection	-–19.6	–12.9	–22.6	–16.6
Si addition	8.0	5.3	5.0	11.0
P deficiency	–4.4	–2.9	–7.4	–1.4
P. plurivora × Si addition	6.1	4.1	3.1	9.2
P deficiency × *P. plurivora*	3.1	2.1	0.1	6.1

CL-confidence limit of the estimate.

Table S4. The impact of experimental factors (P supply, *Phytophthora plurivora* infection and Si addition) on the total root volume per plant (mm^3). The significant coefficients of the 3-way ANOVA model (80 samples; α=0.05) are shown. Full model R^2adj. 0.76, F(7,72)=36.3, p<0.000000

Parameter	Root volume (mm^3 per plant)	t value	-95% CL	+95% CL
Overall mean	1370	51.5	13167	1423
P. plurivora infection	-350	13.2	-403	-297
Si addition	150	5.6	97	203
P deficiency	-144	-5.4	-197	-91
P. plurivora × Si addition	80	3.0	27	133
P deficiency × *P. plurivora*	80	3.0	133	27

CL-confidence limit of the estimate.

Table S5. The impact of experimental factors (P supply, *Phytophthora plurivora* infection and Si addition) on the length of thin roots (diameter 1-2 mm) per plant. The significant coefficients of the 3-way ANOVA model ($\alpha=0.05$) are shown. Full model R^2adj. 0.77; Model $F(7,72)=38.2$, $p<0.0000001$.

Parameter	Length of thin roots (mm per plant)	t value	-95% CL	+95% CL
Overall mean	44.1	58.0	42.6	45.6
P. plurivora infection	−10.8	14.2	−12.3	−9.3
Si addition	4.0	5.3	2.5	5.5
P. plurivora × Si addition	4.0	5.3	2.5	5.5
P deficiency × Si addition	2.1	2.8	0.6	3.6

CL-confidence limit of the estimate.

Table S6: The impact of experimental factors (P supply, *Phytophthora plurivora* infection, and Si addition) on the projected area of thin roots (diameter 1-2 mm) per plant. The significant coefficients of the 3-way ANOVA model ($\alpha=0.05$) are shown. Full model R^2adj. 0.73, $F(7,72)=31.1$, $p<0.0000001$.

Parameter	Projected thin roots area (cm^2 per plant)	t value	-95% CL	+95% CL
Overall mean	6.08	47.8	5.82	6.33
P. plurivora infection	−1.65	−13.0	−1.90	1.39
Si addition	0.53	4.2	0.28	0.79
P deficiency	−0.48	−3.8	−0.73	−0.23
P. plurivora × Si addition	0.44	3.5	0.19	0.69
P deficiency × *P. plurivora*	0.32	2.5	0.07	0.57

CL-confidence limit of the estimate.

Table S7. The effect of treatment factors (P supply, *Phytopthora plurivora* root infection and Si addition) on leaf elemental concentrations in 12-weeks-old oak seedlings. Mean values ± standard deviation for 10 plants per treatment are presented. The same letters in a row (referring to each element) denote that the mean values do not significantly differ among the treatments (Tukey test after 3-way ANOVA for each element, α=0.05).

Leaf elemental concentrations	Treatment							
	P deficiency				Adequate P supply			
	–Phyt–Si	-Phyt+Si	+Phyt–Si	+Phyt+Si	–Phyt–Si	–Phyt+Si	+Phyt–Si	+Phyt+Si
N (%)	1.7±0.3bc	1.8±0.5bc	1.5±0.2ab	1.4±0.2a	1.7 ± 0.1bc	1.8 ± 0.2c	1.4±0.1a	1.5±0.1ab
P (%)	0.08±0.0ab	0.09±0.01bc	0.07±0.01a	0.07±0.01a	0.11±0.01de	0.12±0.0e	0.09±0.0bc	0.10±0.02cd
K (%)	0.58±0.07c	0.51±0.03abc	0.50±0.03ab	0.44±0.06a	0.59 ± 0.06c	0.56±0.04bc	0.57±0.05bc	0.49±0.05ab
Ca (%)	1.3±0.2c	1.1±0.2ab	1.4±0.2c	1.0±0.2a	1.3 ± 0.2c	1.43±0.07c	1.42±0.07c	1.44±0.04c
Mg (%)	0.47±0.09d	0.30±0.08a	0.43±0.06cd	0.34±0.06ab	0.39±0.04bc	0.41±0.06c	0.41±0.07cd	0.34±0.04ab
S (%)	0.152±0.008c	0.14±0.02c	0.12±0.01b	0.05±0.01a	0.15±0.01c	0.16±0.01c	0.11±0.02b	0.18±0.02d
B (mg kg^{-1})	19±5cd	12±3a	16±2bc	22±2d	16 ±2bc	18 ±3bc	20 ±3cd	14 ±3ab
Cu (mg kg^{-1})	2.4 ± 0.6 a	4 ± 1c	1.5±0.4a	4±1c	1.8 ± 0.6 a	1.6 ± 0.6 a	2.0 ± 0.7 a	2.6±0.9ab
Fe (mg kg^{-1})	161±22 b	189±46b	185±36b	110±17a	173 ± 26 b	187 ± 31 b	175 ± 31 b	181±38b
Mn (mg kg^{-1})	764±155 c	477±86ab	662±107bc	455±42a	723 ± 90 c	784±160c	735±119c	806±127c
Zn (mg kg^{-1})	16±2 a	16±3a	18±3a	16±2a	17 ± 3 a	17±3a	16±3a	15±3a
Ni (mg kg^{-1})	0.044±0.001c	0.049±0.003 d	0.035±0.00a	0.039±0.00b	0.053±0.003 e	0.053±0.00e	0.037±0.001ab	0.040±0.001b
Mo (mg kg^{-1})	0.21±0.01c	0.26±0.01d	0.12±0.02a	0.18±0.01b	0.28±0.01 d	0.16±0.01b	0.27±0.02d	0.18±0.01b
Si (%)	0.129±0.009a	0.4±0.2b	0.12±0.02a	0.4 ± 0.2 b	0.11 ± 0.04 a	0.4±0.1b	0.2±0.1a	1.1±0.4c

Table S8. Pairwise comparisons (MRPP test) of leaf ionomes of 12-week-old oak seedlings subjected to the combinations of abiotic (P deficiency) and biotic (*Phytophthora plurivora* root infection) stresses. Element concentrations were adjusted to standard deviate prior to the analysis; Euclidean distance measure was used. Data matrix: 8 treatments (10 plants in each), 14 elements (13 mineral nutrients and Si). T: test statistic (the more negative the value, the stronger the separation of treatments. A: chance-corrected within group agreement (within group homogeneity). Overall T: -30.5, overall A: 0.32, p<0.0000001.

Treatment comparison	T	A	p
−P−Phy−Si *vs* −P−Phyt+Si	−8.0	0.14	0.00003
−P−Phyt−Si *vs* −P+Phyt+Si	−10.7	0.24	0.00001
−P−Phyt−Si *vs* −P+Phyt−Si	−8.7	0.15	0.000009
−P−Phyt−Si *vs* +P−Phyt−Si	−7.6	0.14	0.00002
−P−Phyt-Si *vs* +P−Phyt+Si	−8.2	0.11	0.00008
−P−Phyt−Si *vs* +P+Phyt+Si	−10.2	0.21	0.000005
−P−Phyt−Si *vs* +P+Phyt−Si	−4.4	0.07	0.0023
−P−Phyt+Si *vs* –P+Phyt+Si	−10.2	0.21	0.000009
−P−Phyt+Si *vs* −P+Phyt−Si	−11.0	0.24	0.000005
−P−Phyt+Si *vs* +P−Phyt−Si	−7.9	0.13	0.00002
−P−Phyt+Si *vs* +P−Phyt+Si	−8.7	0.16	0.00002
−P−Phyt+Si *vs* +P+Phyt+Si	−9.6	0.18	0.00001
−P−Phyt+Si *vs* +P+Phyt−Si	−10.4	0.21	0.000005
−P+Phyt+Si *vs* −P+Phyt−Si	−11.7	0.27	0.000005
−P+Phyt+Si *vs* +P−Phyt−Si	−12.2	0.36	0.000006

−P+Phyt+Si *vs* +P−Phyt+Si	−12.3	0.32	0.000006
−P+Phyt+Si *vs* +P+Phyt+Si	−12.1	0.32	0.000006
−P+Phyt+Si *vs* +P+Phyt−Si	−10.9	0.23	0.000006
−P+Phyt-Si *vs* +P−Phyt−Si	−12.2	0.31	0.000004
−P+Phyt−Si *vs* +P−Phyt+Si	−12.0	0.31	0.000004
−P+Phyt−Si *vs* +P+Si−Phyt	−11.4	0.25	0.000005
−P−Si+Phyt *vs* +P+Phyt−Si	−4.9	0.07	0.0007
+P−Phyt-Si *vs* +P−Phyt+Si	−2.2	0.03	0.039
+P−Phyt−Si *vs* +P+Phyt+Si	−11.8	0.28	0.000005
+P-Phyt-Si *vs* +P+Phyt-Si	−11.1	0.23	0.000003
+P−Phyt+Si *vs* +P+Phyt+Si	−11.0	0.24	0.000004
+P−Phyt+Si *vs* +P+Phyt−Si	−10.6	0.24	0.000005
+P+Phyt+Si *vs* +P+Phyt−Si	−10.6	0.21	0.000004
